# Factors associated with knowledge and vaccination intention for human papillomavirus on trans girls by their main caregiver: A cross-sectional study

**DOI:** 10.3389/fimmu.2023.1097449

**Published:** 2023-03-30

**Authors:** Jesus Domínguez-Riscart, Ana-Belen Ariza-Jimenez, Celia Baez-Castillo, Isabel Mateo-Gavira

**Affiliations:** ^1^ Pediatric Department, Hospital Universitario Puerta del Mar, Cádiz, Spain; ^2^ Biomedical Research and Innovation Institute of Cádiz (INiBICA), Cádiz, Spain; ^3^ Pediatric Department, Hospital Universitario Reina Sofia, Córdoba, Spain; ^4^ Instituto Maimónides de Investigación Biomédica de Córdoba (IMIBIC), Córdoba, Spain; ^5^ Endocrinology and Nutrition Department, Hospital Universitario Puerta del Mar, Cádiz, Spain

**Keywords:** transwomen, adolescents, main caregiver, HPV knowledge, human papilloma virus - HPV, vaccination intention

## Abstract

**Introduction:**

Trans women are highly affected by the human papillomavirus (HPV) and are at risk of suffering from HPV-related diseases such as oropharyngeal, anal, penile, or neovaginal neoplasia. HPV vaccination seems to be a good strategy to reduce HPV-related diseases, mainly during the early age before the first sexual intercourse, but only cisgender girls are covered by the National Health Services, while some high-risk groups such as trans girls are not included. Achieving a high vaccination rate is important in the adolescent population, but there are many factors that could affect it, such as lack of knowledge about HPV or fear of side effects by patients and main caregivers. The aim of our study is to analyze the knowledge of trans girls’ main caregivers about HPV-related diseases in the general population and, in particular, in trans women, as well as factors associated with HPV vaccination intention.

**Methods:**

A cross-sectional study was performed with the collaboration of main caregivers of adolescent trans girls, between 9 and 16 years old, assisted in two reference centers’ multidisciplinary Gender Diversity Units. Information was requested through a self-completed questionnaire: HPV-related diseases Knowledge Transwomen questionnaire (HPV-TQ) was elaborated based on a 19-item self-administered questionnaire and score was standardized from 0 to 19 points. Percentage of correct answers was calculated and defined by the group of high scores that showed over 70% correct answers.

**Results:**

A total of 65 main caregivers were included. Almost all main caregivers were mothers with a Caucasian ethnicity. The HPV-TQ average score was 11 (3.7) with an average correct answer of 58.1% (19.6). Only 17/65 (26.1%) of main caregivers were highly knowledgeable in HPV. Of 65 trans girls, 14 were already vaccinated (29.8% of trans girls over 12 years old); 78.5% were not vaccinated and only 21.5% had intentions to be vaccinated. The group with a high score in HPV-TQ had a longer follow-up at the transgender unit, a higher maternal vaccination rate, and a positive family history of HPV-related disease, especially in mothers.

**Conclusion:**

Adolescent trans girls attended to in our units had a low rate and a low intention of vaccination against HPV. Education on and promotion and prevention of transgender HPV-related diseases should probably be implemented to achieve a higher knowledge and vaccination coverage in adolescent trans girls.

## 1 Introduction

Human papillomavirus (HPV) infection is considered the most frequent sexually transmitted infection in the world (1). Several studies suggest a high lifetime probability of HPV acquisition early in life, as reported by a US cohort, in which more than 80% of women and men acquire HPV by the age of 45 years (2). HPV infection can lead to a variety of consequences besides cervical cancer, such as genital warts and oropharyngeal, penile, anal, vulvar, or vaginal cancer (3).

Transgender women are not exempted from HPV risk infection. Indeed, some studies demonstrate a high prevalence of high-risk serotypes and the presence of anogenital warts in adult trans women, such as that of Brown et al. (2016), which shows that up to 96% of trans women have HPV infection, 58.8% of whom were high-risk serotypes and up to 19.1% have anogenital warts (4). Along this line, Jalil et al. (2021) report that up to 77.9% of trans women were infected with HPV, with 60.7% being high-risk serotypes (5). In addition, an American study reports that transsexual women have a higher prevalence of anal HPV infection than men who have sex with men, 88.6% versus 70.9%, respectively (6). Moreover, although the prevalence of neovaginal infection is unknown, five cases of squamous cell carcinoma have been related to HPV infection in the neovagina of transgender women, some of which are at an advanced stage, perhaps due to the low concern about it (7–11).

HPV vaccination programs are mainly designed to protect people from cervical cancer. However, in recent years, scientific evidence suggests that the vaccine may protect against other neoplastic entities (12, 13). Furthermore, the World Health Organization recommends vaccination in adolescents of both genders (14). Main vaccination programs are focused on cisgender female adolescents prior to the first sexual intercourse, approximately 12–14 years old, and high-risk groups, such as those engaged in prostitution, people in contact with HIV, conizated women, and men who have sex with men (15). However, trans women are not included, probably due to a lack of knowledge, which could have a negative impact on their health. In fact, in a survey in which two trans women were recruited, only one received recommendation for HPV vaccination (16). Such data suggest missed opportunities to address the need for HPV vaccination among transgender people.

Achieving a high vaccination rate is important in the adolescent population, but many factors contribute to vaccination refusal by adolescents and their families (17). Many studies show the impact of acceptance or denial of the vaccination according to the opinion of the main caregiver of adolescents. Some factors, such as efficacy, the fear related to vaccine safety, or a poor knowledge about risk and consequences of HPV infection, are reported to be crucial when choosing to vaccinate or not (18, 19).

Currently, there is no published study that focused on the feelings and knowledge of main caregivers of trans adolescents, in order to improve the adherence to vaccination. Our aim is to determine the main trans women caregivers’ knowledge about HPV infection and risk, and to detect which factors could influence the intention of vaccination.

## 2 Materials and methods

A cross-sectional study was conducted based on interviews of main caregivers of adolescents under 16 years old followed up in two reference centers with a multidisciplinary Sex Reassignment Treatment Unit: Hospital Puerta del Mar (Cádiz, Spain) and Hospital Universitario Reina Sofia (Cordoba, Spain). Inclusion criteria were as follows: main caregivers of transgender women between 9 and 16 years old who were involved in Sex Reassignment Treatment Units between January 2022 and July 2022, completed the questionnaire, and signed the consent form.

The survey instrument was HPV-related diseases Knowledge Transwomen questionnaire (HPV-TQ), a 19-item self-administered questionnaire with True–False forced-response questions based on a validated survey but adapted to the transgender population (20). It covers basic knowledge of HPV infection as its characteristics, including transmission, viral properties, treatment, prevalence, vaccination, perception of sexual risk behavior, and specific risk in trans women. The content, readability, and comprehensiveness of the questionnaire were reviewed by clinicians and health educators. Answers were evaluated. Correct answers were scored as 1 point, and wrong answers received 0 points. The final score was the result of the sum of all points, ranging from 0 to 19. This score was converted into percentage, standardized from 0 to 100. A score equal to and/or over 70% was classified as high HPV knowledge. HPV vaccination status and demographic and clinical data were collected from the clinical history of trans girls.

All participants provided a written informed consent before data collection in accordance with the Declaration of Helsinki, and researchers guaranteed the anonymity of data. The study was approved by the Ethics Committee of Puerta del Mar University Hospital (ethical approval code number PEIBA 0474-N-22).

Statistical analysis was performed with SPSS version 23.0 (IBM, Armonk, NY, USA). Descriptive statistics were calculated for all variables. Continuous measures were summarized by using mean and standard deviation, and dichotomous measures were summarized by using proportions. Student’s *t*-test was performed to compare continuous variables and *χ* ^2^ was used when proportions are compared. *p* < 0.05 was considered statistically significant.

## 3 Results

### 3.1 Clinical and sociodemographic characteristics

A total of 65 main trans women adolescent caregivers were included. The average age of trans girls was 13.3 (2.7) years, and the mean age of those visiting the unit was 9.1 (3.4) years old; the main ethnic group was Caucasian (62/65, or 95.4%). Currently, puberal blockage and crossed hormone therapy have been started in 41/65 (63.1%) and 26/65 (40%) women, respectively. Comparing this variable within low- and high-HPV knowledge groups, we only found longer follow-up in the high-HPV knowledge group in comparison with its low counterpart, with differences being statistically significant. Almost all main caregivers were the trans girls’ mothers, whose mean age was 44.1 (5.6) years old. The most frequent educational level was general certificate of secondary education in 27/65 caregivers (41.5%), followed by university studies with 17/65 caregivers (26.2%); 44/65 caregivers (67.7%) were married. No differences were found in the sociodemographic characteristics of the main caregivers when they were compared by HPV knowledge level. All data are shown in **Table 1**.

**Table 1:** Clinical and sociodemographic characteristics of adolescent trans girls and their main caregiver.

Variable	Total (*N* = 65)	Main caregiver HPV-related knowledge		*p*-value
		Low (*N* = 48)	High (*N* = 17)	
Characteristics of adolescent trans girls				
Current age (years)	13.3 (2.7)	13.3 (2.8)	13.3 (2.7)	0.56
12 years old or more	47 (72.3)	13 (76.5)	34 (70.8)	0.65
Ethnic group				
Caucasian	62 (95.4)	47 (97.9)	15 (88.2)	0.102
Gipsy	3 (4.6)	1 (2.1)	2 (11.8)	
First visit at the unit age (years)	9.1 (3.4)	9.5 (3.4)	8.2 (3.4)	0.95
Pubertal blockage				
No	24 (36.9)	20 (41.7)	4 (23.5)	0.18
Yes	41 (63.1)	28 (58.3)	13 (76.5)	
Pubertal blockage age at onset (years)	12.7 (1.89)	14.0 (1.2)	13.9 (1.7)	0.19
Crossed hormone therapy				
No	39 (60)	29 (60.4)	10 (58.8)	0.90
Yes	26. (40)	19 (39.4)	7 (41.2)	
Crossed hormone therapy age at onset (years)	15.3 (1.3)	15.4 (1.2)	15.1 (1.1)	0.83
Characteristics of the main caregiver				
Age (years)	44.1 (5.6)	43.4 (5.7)	44.5 (5.8)	0.64
Sex				
Female	64 (98.5)	47 (97.9)	17 (100)	0.87
Male	1 (1.5)	1 (2)	0 (0)	
Relationship with the trans adolescent				
Mother	63 (96.8)	47 (97.9)	16 (94.2)	0.56
Father	1 (1.5)	1 (2.1)	0 (0)	
Grandmother	1 (1.5)	0 (0)	1 (5,9)	
Education level				
University	17 (26.2)	12 (25)	5 (29.4)	0.68
Vocational training	14 (21.5)	9 (18.8)	5 (29.4)	
General certificate of secondary education	27 (41.5)	21 (43.8)	6 (35.3)	
Elementary or less	7 (10.8)	6 (12.4)	1 (5.9)	
Marital status				
Married	44 (67.7)	33 (68.7)	11 (64.7)	0.75
Single	21 (32.3)	15 (31.3)	6 (35.3)	

### 3.2 Adolescent trans girls’ vaccination status and intentions

Of 65 girls, 14 were already vaccinated, all over 12 years old. They represent 21.5% of the total sample and 29.8% of the total girls over 12 years old in the sample, which is the recommended age for vaccination in cisgender girls. Only 1/7 (7.1%) of all vaccinated trans girls was vaccinated to prevent transgirl HPV-related risk, while the rest were vaccinated by following a cisgender girl program. The non-vaccinated rate was high, 51/65 (78.5%), and only 11/51 (21.5%) intended to be vaccinated. Similar data are shown in groups under and over 12 years old, 22.2% and 14.8%, respectively; thus, no differences were found. Data are shown in **Table 2**. Although we did not find differences in the rate of vaccination, **Table 3** shows higher intention of vaccination in the high-HPV knowledge group in comparison to the low-HPV knowledge group, 53.8% and 10.5%, respectively.

**Table 2:** Vaccination and intention of vaccination in trans girls according to age.

Variable	Total (*N* = 65)	Age group		*p*-value
		Under 12 years old (*N* = 18)	Over 12 years old (*N* = 47)	
Already vaccinated	14 (21.5)	0 (0)	14 (29.8)	0.009
Vaccination due to risk in trans girls	1 (7.1)	0 (0)	1 (7.1)	0.003
Just followed the cis adolescent program	13 (92.9)	0 (0)	13 (92.9)	
Non-vaccinated	51 (78.5)	18 (76.5)	33 (79.2)	
Vaccination intention	11 (21.6)	4 (22.2)	7 (14.8)	0.96
No intention of vaccination	40 (78.4)	14(89.5)	26 (55.6)	

**Table 3:** HPV-related variables according to the main caregiver’s HPV knowledge.

Variable	Total (*N* = 65)	Main caregiver score HPV-TQ		*p*-value
		Low (*N* = 48)	High (*N* = 17)	
Score on HPV-TQ	11 (3.7)	9.5 (3)	15.2 (1.5)	0.01
Score on HPV-TQ in percentage	58.1 (19.6)	50.2 (16.1)	80.4 (8)	0.01
Score HPV-TQ > 70%	17 (26.1)	0 (0)	17 (100)	0.001
Follow-up in the transgender unit (years)	2.8 (2.4)	2.3 (1.8)	4.1 (3.4)	0.002
Vaccination in trans girls				
Already vaccinated	14 (21.5)	10 (20.8)	4 (23.5)	0.81
Non-vaccinated	51 (78.5)	38 (79.2)	13 (76.5)	
Vaccination intention	11 (16.9)	4 (10.5)	7 (53.8)	0.01
No intention of vaccination	40 (61.5)	34 (89.5)	6 (46.2)	
Main caregiver knew that HPV is an ITS				
No	22 (33.8)	19 (39.6)	3 (17.6)	0.100
Yes	43 (66.2)	29 (60.4)	14 (82.4)	
Knowledge about HPV vaccination				
No	8 (12.3)	8 (16.7)	0 (0)	0.07
Yes	57 (87.7)	40 (83.3)	17 (100)	
HPV family history				
HPV family vaccination				
No	45 (69.2)	35 (72.9)	10 (58.8)	0.27
Yes	20 (30.8)	13 (27.1)	7 (41.2)	
Maternal vaccination	4 (6.2)	0 (0)	4 (23.5)	0.001
Paternal vaccination	0 (0)	0 (0)	0 (0)	–
Sister vaccination	19 (29.2)	13 (27.1)	6 (35.3)	0.52
Brother vaccination	0 (0)	0 (0)	0 (0)	–
HPV family-related disease				
No	58. (89.2)	46 (95.8)	12 (70.6)	0.05
Yes	7(10.8)	2 (4.1)	5 (29.4)	
Maternal HPV-related disease	6 (85.7)	81 (50)	5 (100)	0.05
Other relatives	1 (14.3)	1 (50)	0 (0)	

### 3.3 HPV-related knowledge

**Figure 1:**
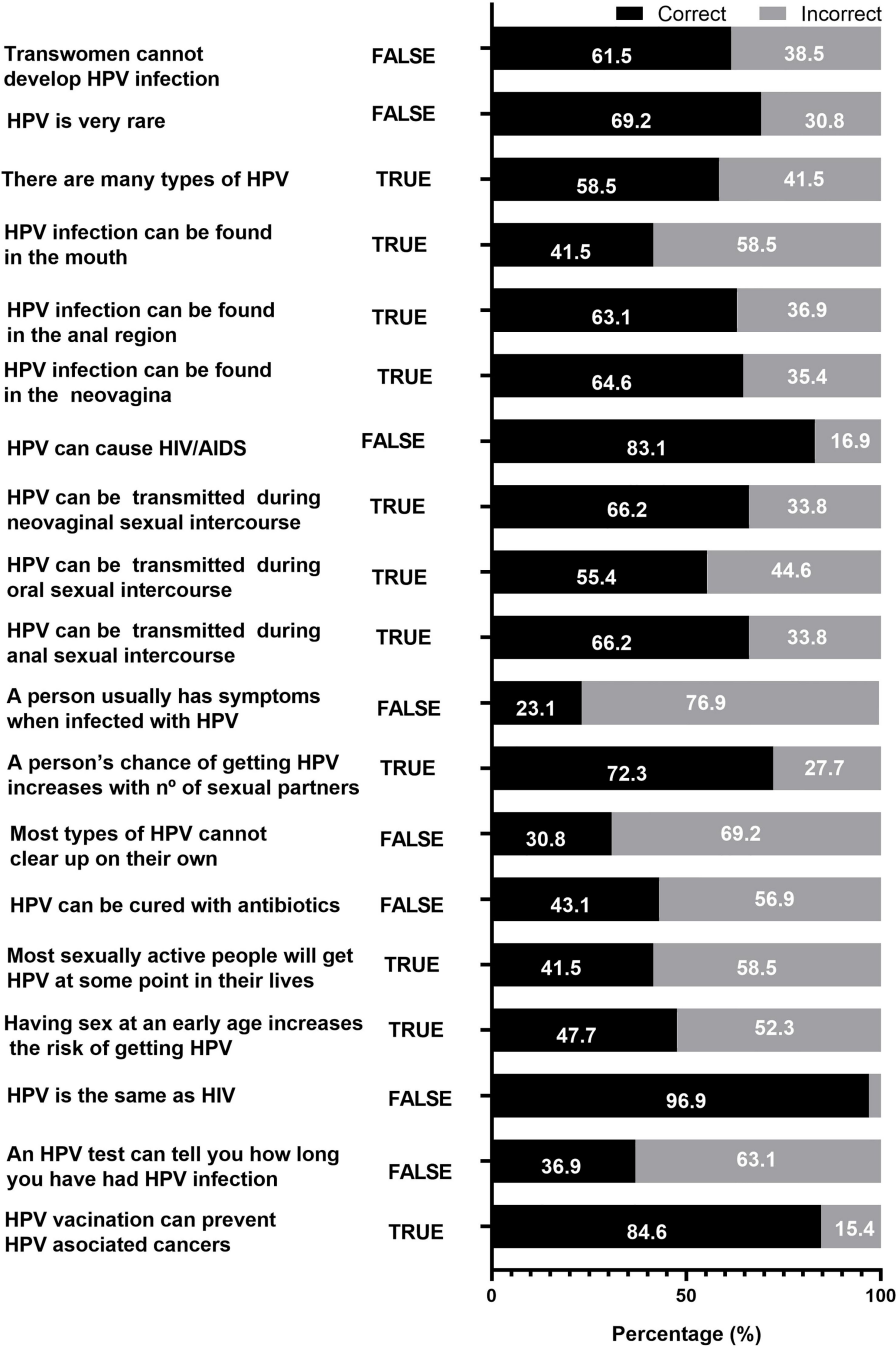
HPV-TQ questions and response of trans girls’ main caregivers.

The HPV-TQ mean score was 11 (3.7), and the mean percentage of correct answers was 58.1% (19.6). Only 17/65 (26.1%) main caregivers were highly knowledgeable in HPV. **Figure 1** shows the percentage of correct and incorrect answers to each question. The highest rates were found in the two questions about differentiation with human immunodeficiency virus (HIV): “HPV can cause AIDS” and “HPV is the same as HIV”, with 83.1% and 96.9%, respectively. Also, we found a correct answer of 84.6% to the question “HPV vaccination can prevent HPV-associated cancers”. The lowest rates were found in questions related to clinical presentation, physiopathology, diagnosis, or prevalence, such as “A person usually has symptoms when is infected by HPV” (23.1%); “Most types of HPV cannot clear up on their own” (30.8%); “Most sexually active people will get HPV at some point in their lives” (41.5%); “HPV infection can be found in the mouth” (41.5%); or “HPV can be cured by antibiotics” (43.1%). Questions about transgender risk of HPV contagion such as “Trans women cannot develop HPV infections”, “HPV infection can be found in the neovagina”, and “HPV can be transmitted by neovaginal intercourse” had correct responses of 61.5%, 64.6%, and 66.2%, respectively.

### 3.4 Related factors’ influence on HPV-related knowledge

**Table 3** summarizes the personal and family history of HPV-related disease. We found that the group with a high score in HPV-TQ had a longer follow-up at the transgender units, a higher proportion in maternal vaccination, and a positive family history of HPV-related disease, especially in mothers. History of trans girls’ or trans girls’ sisters’ vaccination did not have statistically significant differences. Although it seemed that we found a higher rate of main caregivers who knew that HPV was an ITS and who knew about the HPV vaccine, no statistically significant differences were found.

## 4 Discussion

Gay, bisexual, and other men who have sex with men and transgender women are disproportionately affected by HPV. HPV vaccination is most effective when given before exposure to HPV through sexual activity. Vaccination is routinely recommended in some countries such as USA for adolescents at age 11–12 years, with catch-up vaccination through age 26 years. However, Meites et al. (2022) showed a median age at first dose of 19 years and a median age at first sexual intercourse of 17 years; 493 (73.3%) reported that their age at first dose was older than their age at first sexual intercourse (21). Spain probably had a similar situation, which could explain the low vaccination rate in the recommended age group. National vaccination surveys reported that our region, Andalusia (Spain), has the lowest vaccination rate in the country. Only 75% of adolescent cisgender girls had the first vaccine dose, and 59.9% had the second one (22), but adolescent trans girls have lower rates: only 28.9% of those over 12 years old have been vaccinated. The rate in our study might not be considered the real prevalence, because not all trans adolescents of the region were evaluated in our study, but the rate could be representative of the population. Also, we observed a low vaccination intention; only 16.9% of trans girl main caregivers considered the vaccination important for their minor children. Similar data were reported in studies focused on main caregivers of adolescent cisgender boys (23, 24), cisgender adult men (25), and men who have sex with men, probably because of the lack of knowledge of the risk for oral, penile, and anal cancer (26), and because HPV infection was considered an issue only for cisgender girls or women, due to health marketing and campaign efforts to achieve higher vaccination in this population (24).

The group of main caregivers who were highly knowledgeable about HPV showed a significantly longer follow-up at our specialized units, which might be explained by the fact that related information about risk of HPV in trans girls could be provided by gender diversity unit specialists who are knowledgeable about the importance of HPV vaccination. Vaccination in our region is programmed in primary care centers. A study in Andalusian primary care revealed that low knowledge of professionals leads to doubts in vaccination as well as unclear information provided to patients and families (27). In addition, professionals need to be sensitive to the specific health problems of trans women, such as HPV-related diseases, in order to reduce stigma and healthcare barriers (28). It is important for professionals to transmit reliable information to main caregivers and adolescent trans girls.

A similar situation has been experienced in other countries. In a 2014 survey conducted in USA involving rural participants enrolled online that included 23 transgender women who were age-eligible for HPV vaccination, only one reported receiving a healthcare provider recommendation for HPV vaccination, and only one reported ever receiving ≥1 dose of HPV vaccine. Such data suggest missed opportunities to address the need for HPV vaccination among transgender people as well as in our country (29). Increasing both routine and catch-up vaccination will improve coverage among transgender women (21).

Several studies revealed the main predictors of vaccination, which included, apart from healthcare provider recommendation, younger age, having a healthcare visit in the past year, receiving other recommended vaccinations, and living with HIV (21, 29, 30). Another interesting point is reported by Pho et al. (2022), which is associated with online health information seeking and HPV vaccination (31). Decreased reporting of HPV vaccination among trans people after searching for vaccine information online suggests vaccine hesitancy, which may potentially be related to the quality of online content. Increased reporting of vaccination after using social media may be related to peer validation. Future studies should investigate potential deterrents to HPV vaccination in online health information to enhance its effectiveness and further explore which aspects of social media might increase vaccine uptake among trans people.

Although other studies revealed higher knowledge in vaccinated adolescents’ parents (32), we did not observe this effect, because almost all adolescent trans girls in our study were vaccinated without any specific motivation; they just followed the cis girl program. We observed that a previous history of HPV disease in the first-line family is associated with a higher knowledge score and higher intention of vaccination. Other studies found an inverse correlation between knowledge and age of main caregivers (33), but our data did not support this. Moreover, educational level is reported as important. Higher HPV knowledge has been related to higher educational attainment (34); however, our study showed equally distributed educational levels. Gender and ethnicity influence could not be analyzed in our study because almost all participants were Caucasian and women.

Mean knowledge in our study was acceptable but some questions had a low proportion of correct responses such as those related to clinical presentation, natural history of HPV infection, diagnosis method, and treatment. This could reflect ignorance about basic aspects of HPV infection. These findings are similar to a study involving the main caregivers of adolescent cis boys from UK. Firstly, in the question “HPV can be cured by antibiotics” in our study, only 43.1% of main caregivers answered correctly, compared to 48.5% in the adolescent cis boys’ main caregiver study (23). Also, the question “Most sexually active people will get HPV at some point in their lives” had 41.5% and 32.3% correct responses, respectively (23). Interestingly, oropharyngeal infection by HPV is unknown for many main caregivers in our study; only 41.5% knew that it could be found in the mouth, lower than that reported by the adolescent cis boys’ main caregiver survey study (49.5%) (23) and that reported by a study in an adult population in USA (49.3%) (20).

The strength of our study resides in the fact that limited data about HPV in the trans population are available (35). In fact, in USA, database searches and manual searching yielded 843 citations. After screening, eight articles were retained in the review. Seven were cross-sectional studies and one was a qualitative focus group. The low proportion of transgender participants in the retained studies highlights a gap in knowledge about HPV vaccination among this population. Future studies of HPV vaccination should recruit trans people to better represent their perspectives and data (31). There are some limitations in our study, such as having a small sample size and the low diversity in caregivers, because most of them were mothers. Two main caregivers refused to participate. Thus, our results could be susceptible to selection bias because the main caregivers who participated might be more health conscious and curious about HPV. In that case, HPV knowledge could be lower if this is true.

## 5 Conclusions

This study showed a low vaccination proportion, only 21.5%. Also, 21.5% of main caregivers of unvaccinated adolescent trans girls intend to be vaccinated. We observe that intention of vaccination is related to HPV knowledge. Knowledge in HPV was related to a previous family history of HPV-related disease, maternal HPV vaccination, and longer time of follow-up in our units. The results should encourage the development and implementation of health education programs that discuss the HPV vaccine and its benefits for trans women at early age. General recognition and approval of vaccination programs for trans women by health international institutions and campaigns focused on main caregivers to increase knowledge and awareness about the virus and vaccination can help raise the vaccination rate and improve health equality.

## Data availability statement

The original contributions presented in the study are included in the article/supplementary material. Further inquiries can be directed to the corresponding author.

## Ethics statement

The studies involving human participants were reviewed and approved by COMITÉ DE ÉTICA DE LA INVESTIGACIÓN DE CÁDIZ. Written informed consent to participate in this study was provided by the participants’ legal guardian/next of kin.

## Author contributions

JD-R and IM-G conceptualized and designed the study, contributed to the clinical recruitment of participants, supervised the database, performed statistical analyses, drafted the initial manuscript, revised it, and wrote the final version. CB-C performed the dataset and reviewed and revised the manuscript. A-BA-J reviewed the study design, contributed to the clinical recruitment of participants, and reviewed and revised the manuscript for the final version. All authors contributed to the article and approved the submitted version.
